# (*Z*)-4-(3-Fluoro­phen­yl)-1-(5-nitro-2-oxo­indolin-3-yl­idene)thio­semicarbazide

**DOI:** 10.1107/S1600536812005053

**Published:** 2012-02-10

**Authors:** Humayun Pervez, Nazia Manzoor, Muhammad Yaqub, M. Nawaz Tahir

**Affiliations:** a Bahauddin Zakariya University, Department of Chemistry, Multan 60800, Pakistan; b University of Sargodha, Department of Physics, Sargodha, Pakistan

## Abstract

In the title compound, C_15_H_10_FN_5_O_3_S, an intra­molecular N—H⋯N hydrogen bond generates an *S*(5) ring, whereas N—H⋯O and C—H⋯S inter­actions complete *S*(6) ring motifs. The dihedral angle between the isatin ring system and the fluoro­benzene ring is 5.96 (6)° and the complete mol­ecule is close to planar (r.m.s. deviation for all the non-H atoms = 0.112 Å). In the crystal, mol­ecules are linked by N—H⋯O hydrogen bonds to form *C*(8) chains along the [100] direction and C—H⋯O inter­actions are also observed.

## Related literature

For background to isatin derivatives, see: Pervez *et al.* (2010 ▶); Pervez, Ramzan *et al.* (2011 ▶); Pervez, Saira *et al.* (2011 ▶). For related structures, see: Pervez *et al.* (2009 ▶); Ramzan *et al.* (2010 ▶). For graph-set notation, see: Bernstein *et al.* (1995 ▶).
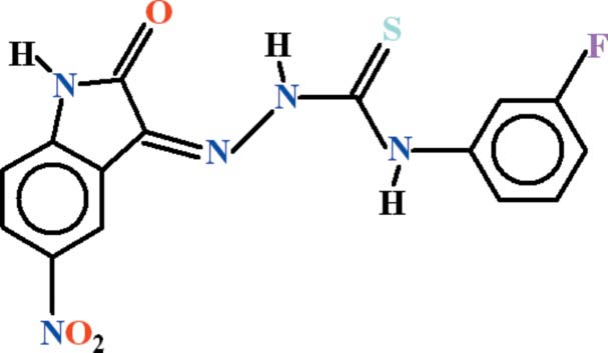


## Experimental

### 

#### Crystal data

C_15_H_10_FN_5_O_3_S
*M* *_r_* = 359.34
Orthorhombic, 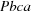

*a* = 18.2485 (5) Å
*b* = 8.8043 (2) Å
*c* = 18.6913 (5) Å
*V* = 3003.04 (13) Å^3^
*Z* = 8
Mo *K*α radiation
μ = 0.26 mm^−1^
*T* = 296 K
0.30 × 0.25 × 0.20 mm

#### Data collection

Bruker Kappa APEXII CCD diffractometer
Absorption correction: multi-scan (*SADABS*; Bruker, 2005 ▶) *T*_min_ = 0.957, *T*_max_ = 0.966
53231 measured reflections
3728 independent reflections
3101 reflections with *I* > 2σ(*I*)
*R*_int_ = 0.027

#### Refinement

*R*[*F* ^2^ > 2σ(*F* ^2^)] = 0.037
*wR*(*F* ^2^) = 0.102
*S* = 1.06
3728 reflections
226 parameters
H-atom parameters constrained
Δρ_max_ = 0.19 e Å^−3^
Δρ_min_ = −0.28 e Å^−3^

### 

Data collection: *APEX2* (Bruker, 2007 ▶); cell refinement: *SAINT* (Bruker, 2007 ▶); data reduction: *SAINT*; program(s) used to solve structure: *SHELXS97* (Sheldrick, 2008 ▶); program(s) used to refine structure: *SHELXL97* (Sheldrick, 2008 ▶); molecular graphics: *ORTEP-3 for Windows* (Farrugia, 1997 ▶) and *PLATON* (Spek, 2009 ▶); software used to prepare material for publication: *WinGX* (Farrugia, 1999 ▶) and *PLATON*.

## Supplementary Material

Crystal structure: contains datablock(s) global, I. DOI: 10.1107/S1600536812005053/hb6630sup1.cif

Structure factors: contains datablock(s) I. DOI: 10.1107/S1600536812005053/hb6630Isup2.hkl

Supplementary material file. DOI: 10.1107/S1600536812005053/hb6630Isup3.cml

Additional supplementary materials:  crystallographic information; 3D view; checkCIF report

## Figures and Tables

**Table 1:** Hydrogen-bond geometry (Å, °)

*D*—H⋯*A*	*D*—H	H⋯*A*	*D*⋯*A*	*D*—H⋯*A*
N1—H1⋯N3	0.86	2.18	2.6166 (17)	112
N2—H2*A*⋯O1	0.86	2.06	2.7483 (16)	136
N4—H4*A*⋯O3^i^	0.86	2.26	3.0073 (17)	146
C2—H2⋯S1	0.93	2.49	3.1674 (17)	130
C4—H4⋯O1^ii^	0.93	2.39	3.281 (2)	161
C6—H6⋯O2^iii^	0.93	2.56	3.392 (2)	149
C12—H12⋯O2^iii^	0.93	2.59	3.470 (2)	158

Symmetry codes: (i) 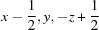; (ii) 
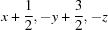
; (iii) 
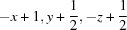
.

**Fig. 1.**
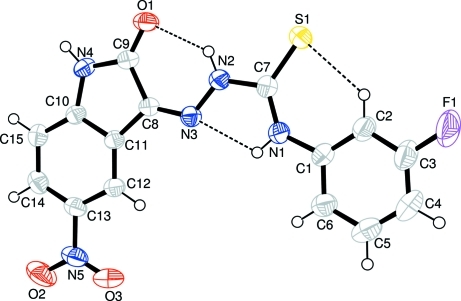
View of the title compound with displacement ellipsoids drawn at the 50% probability level. The dotted lines indicate the intra-molecular H-bondings.

**Fig. 2.**
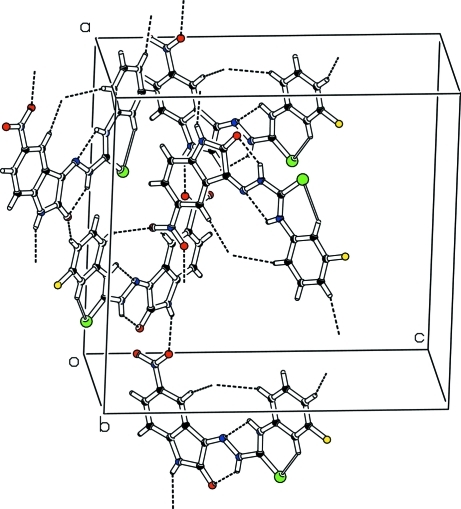
Packing diagram of the title compound (PLATON: Spek, 2009) showing that molecules form one dimensional polymeric chains along [100] and are interlinked.

## supplementary crystallographic information

### Comment

In continuation of our earlier studies on some isatins-thiosemicarbazones (Pervez *et al.* 2010; Pervez, Ramzan *et al.*, 2011; Pervez, Saira *et al.*, 2011), we report herein the structure and synthesis of the title compound (I), (Fig. 1). When compared with the crystal structures of 1-(5-nitro-2-oxoindolin-3-ylidene)-4-*o*-tolylthiosemi carbazide (II) (Pervez *et al.*, 2009) and 4-(3-fluorophenyl)-1-(2-oxoindolin-3-yl-idene)thiosemicarbazides (III) (Ramzan *et al.*, 2010), it can be noticed that it differs from (II) by the presence of fluoro function at position-3 instead of methyl at position-2 of the phenyl ring attached to N of the thiosemicarbazone moiety and from (III) by the presence of nitro substituent at position-5 of the isatin scaffold.

In (I) the fluoro-phenyl group A (C1–C6/F1), thiosemicarbazone moiety B (N1/C7/S1/N2/N3) and 5-nitro-1,3-dihydro-2*H*-indol-2-one group C (C8–C15/N3–N5/O1–O3) are planar with r. m. s. deviations of 0.0019, 0.0245 and 0.0071 Å, respectively. The dihedral angle between A/B, A/C and B/C is 8.67 (7)°, 5.96 (6)° and 4.35 (5)°, respectively. Due to intramolecular H-bondings of N—H···O, N—H···N and C—H···S types (Table 1, Fig. 1), S(6), S(5) and S(6) ring motifs (Bernstein *et al.*, 1995), respectively are formed. The molecules form C(8) chains (Bernstein *et al.*, 1995) due to intermolecular H-bondings of N—H···O type (Table 1, Fig. 2) along [100] direction, where O-atom is of nitro and N–H is of 5-nitro-1,3-dihydro-2*H* -indol-2-one. The polymeric chains are connected throuh C—H···O bonds to form three-dimensional network (Table 1, Fig. 2).

### Experimental

To a hot solution of 5-nitroisatin (0.48 g, 2.5 mmol) in 50% aqueous ethanol (30 ml) containing a catalytic quantity (3–4 drops) of glacial acetic acid was added 3-fluorophenythiosemicarbazide (0.46 g, 2.5 mmol) dissolved in ethanol (10 ml) and the reaction mixture was refluxed for 2 h. The orange crystalline solid formed during refluxing was collected by suction filtration. Thorough washing with hot aqueous ethanol provided the title compound (I) in pure form (0.77 g, 86%), m.p. 511 K. Orange prisms were grown in ethyl acetate-petroleum ether by diffusion method.

### Refinement

The H-atoms were positioned geometrically (N–H = 0.86 Å, C–H = 0.93 Å) and refined as riding with *U*_iso_(H) = x*U*_eq_(C, N), where x = 1.2 for all H-atoms.

### Crystal data

**Table:**

C_15_H_10_FN_5_O_3_S	*F*(000) = 1472
*M**_r_* = 359.34	*D*_x_ = 1.590 Mg m^−^^3^
Orthorhombic, *P**b**c**a*	Mo *K*α radiation, λ = 0.71073 Å
Hall symbol: -P 2ac 2ab	Cell parameters from 3101 reflections
*a* = 18.2485 (5) Å	θ = 2.2–28.3°
*b* = 8.8043 (2) Å	µ = 0.26 mm^−^^1^
*c* = 18.6913 (5) Å	*T* = 296 K
*V* = 3003.04 (13) Å^3^	Prism, orange
*Z* = 8	0.30 × 0.25 × 0.20 mm

### Data collection

**Table:**

Bruker Kappa APEXII CCD diffractometer	3728 independent reflections
Radiation source: fine-focus sealed tube	3101 reflections with *I* > 2σ(*I*)
Graphite monochromator	*R*_int_ = 0.027
Detector resolution: 7.50 pixels mm^-1^	θ_max_ = 28.3°, θ_min_ = 2.2°
ω scans	*h* = −24→21
Absorption correction: multi-scan (*SADABS*; Bruker, 2005)	*k* = −8→11
*T*_min_ = 0.957, *T*_max_ = 0.966	*l* = −24→24
53231 measured reflections	

### Refinement

**Table:**

Refinement on *F*^2^	Primary atom site location: structure-invariant direct methods
Least-squares matrix: full	Secondary atom site location: difference Fourier map
*R*[*F*^2^ > 2σ(*F*^2^)] = 0.037	Hydrogen site location: inferred from neighbouring sites
*wR*(*F*^2^) = 0.102	H-atom parameters constrained
*S* = 1.06	*w* = 1/[σ^2^(*F*_o_^2^) + (0.045*P*)^2^ + 1.1466*P*] where *P* = (*F*_o_^2^ + 2*F*_c_^2^)/3
3728 reflections	(Δ/σ)_max_ < 0.001
226 parameters	Δρ_max_ = 0.19 e Å^−^^3^
0 restraints	Δρ_min_ = −0.28 e Å^−^^3^

### Special details

**Table:**

Geometry. Bond distances, angles *etc*. have been calculated using the rounded fractional coordinates. All su's are estimated from the variances of the (full) variance-covariance matrix. The cell e.s.d.'s are taken into account in the estimation of distances, angles and torsion angles
Refinement. Refinement of *F*^2^ against ALL reflections. The weighted *R*-factor *wR* and goodness of fit *S* are based on *F*^2^, conventional *R*-factors *R* are based on *F*, with *F* set to zero for negative *F*^2^. The threshold expression of *F*^2^ > σ(*F*^2^) is used only for calculating *R*-factors(gt) *etc*. and is not relevant to the choice of reflections for refinement. *R*-factors based on *F*^2^ are statistically about twice as large as those based on *F*, and *R*- factors based on ALL data will be even larger.

### Fractional atomic coordinates and isotropic or equivalent isotropic displacement parameters (Å^2^)

**Table:**

	*x*	*y*	*z*	*U*_iso_*/*U*_eq_	
S1	0.23197 (2)	0.67952 (5)	−0.04002 (2)	0.0468 (1)	
F1	0.41277 (8)	1.02225 (17)	−0.14952 (9)	0.1015 (6)	
O1	0.14958 (6)	0.37037 (13)	0.13575 (6)	0.0491 (4)	
O2	0.50290 (8)	0.02530 (18)	0.34644 (8)	0.0759 (5)	
O3	0.53143 (7)	0.1873 (2)	0.26498 (9)	0.0811 (6)	
N1	0.36565 (7)	0.62197 (15)	0.01839 (7)	0.0421 (4)	
N2	0.26518 (6)	0.51166 (14)	0.06902 (6)	0.0388 (3)	
N3	0.31021 (6)	0.43761 (13)	0.11392 (6)	0.0361 (3)	
N4	0.19541 (6)	0.21196 (13)	0.22400 (7)	0.0379 (3)	
N5	0.48641 (8)	0.11444 (18)	0.29912 (8)	0.0531 (4)	
C1	0.41226 (8)	0.72152 (17)	−0.01991 (8)	0.0400 (4)	
C2	0.38746 (9)	0.8269 (2)	−0.06921 (10)	0.0535 (5)	
C3	0.43845 (11)	0.9188 (2)	−0.10154 (11)	0.0600 (6)	
C4	0.51147 (11)	0.9129 (2)	−0.08870 (11)	0.0608 (6)	
C5	0.53538 (10)	0.8073 (2)	−0.03948 (10)	0.0599 (6)	
C6	0.48660 (9)	0.7113 (2)	−0.00505 (9)	0.0493 (5)	
C7	0.29264 (8)	0.60528 (15)	0.01605 (7)	0.0363 (4)	
C8	0.28008 (7)	0.34713 (15)	0.15985 (7)	0.0329 (3)	
C9	0.20008 (7)	0.31556 (15)	0.16965 (8)	0.0358 (4)	
C10	0.26468 (7)	0.17306 (15)	0.24998 (7)	0.0336 (3)	
C11	0.31818 (7)	0.25505 (15)	0.21213 (7)	0.0324 (3)	
C12	0.39169 (7)	0.23766 (16)	0.22778 (7)	0.0365 (4)	
C13	0.40880 (8)	0.13552 (17)	0.28157 (8)	0.0402 (4)	
C14	0.35672 (9)	0.05368 (18)	0.31899 (8)	0.0445 (5)	
C15	0.28301 (8)	0.07168 (17)	0.30351 (8)	0.0412 (4)	
H1	0.38771	0.56263	0.04792	0.0505*	
H2	0.33789	0.83526	−0.08008	0.0643*	
H2A	0.21856	0.50064	0.07329	0.0466*	
H4	0.54402	0.97735	−0.11205	0.0730*	
H4A	0.15495	0.17542	0.24013	0.0455*	
H5	0.58513	0.80035	−0.02920	0.0719*	
H6	0.50361	0.64038	0.02787	0.0592*	
H12	0.42774	0.29165	0.20349	0.0438*	
H14	0.37122	−0.01361	0.35464	0.0534*	
H15	0.24724	0.01765	0.32818	0.0494*	

### Atomic displacement parameters (Å^2^)

**Table:**

	*U*^11^	*U*^22^	*U*^33^	*U*^12^	*U*^13^	*U*^23^
S1	0.0420 (2)	0.0503 (2)	0.0480 (2)	−0.0007 (2)	−0.0056 (2)	0.0107 (2)
F1	0.0892 (9)	0.0919 (9)	0.1234 (12)	0.0074 (8)	0.0314 (9)	0.0678 (9)
O1	0.0316 (5)	0.0552 (7)	0.0604 (7)	0.0046 (5)	−0.0063 (5)	0.0088 (5)
O2	0.0683 (9)	0.0887 (10)	0.0707 (9)	0.0296 (8)	−0.0231 (7)	0.0079 (8)
O3	0.0348 (6)	0.1214 (14)	0.0871 (10)	0.0002 (7)	−0.0130 (7)	0.0148 (10)
N1	0.0375 (6)	0.0419 (7)	0.0469 (7)	0.0005 (5)	0.0005 (5)	0.0107 (5)
N2	0.0337 (6)	0.0396 (6)	0.0432 (6)	−0.0012 (5)	−0.0009 (5)	0.0070 (5)
N3	0.0362 (6)	0.0333 (6)	0.0389 (6)	0.0002 (5)	−0.0010 (5)	0.0017 (5)
N4	0.0283 (5)	0.0390 (6)	0.0464 (6)	−0.0019 (5)	0.0049 (5)	0.0012 (5)
N5	0.0425 (7)	0.0670 (9)	0.0498 (7)	0.0149 (7)	−0.0125 (6)	−0.0093 (7)
C1	0.0388 (7)	0.0389 (7)	0.0424 (7)	−0.0022 (6)	0.0064 (6)	−0.0028 (6)
C2	0.0431 (8)	0.0528 (10)	0.0647 (10)	0.0002 (7)	0.0091 (8)	0.0175 (8)
C3	0.0615 (11)	0.0509 (10)	0.0677 (11)	−0.0007 (8)	0.0196 (9)	0.0155 (8)
C4	0.0591 (10)	0.0562 (10)	0.0672 (11)	−0.0165 (9)	0.0247 (9)	−0.0065 (9)
C5	0.0416 (9)	0.0712 (12)	0.0670 (11)	−0.0132 (8)	0.0075 (8)	−0.0133 (10)
C6	0.0422 (8)	0.0554 (10)	0.0504 (8)	−0.0035 (7)	−0.0001 (7)	−0.0041 (7)
C7	0.0403 (7)	0.0310 (6)	0.0376 (7)	−0.0005 (5)	0.0017 (5)	−0.0013 (5)
C8	0.0294 (6)	0.0326 (6)	0.0368 (6)	0.0004 (5)	−0.0009 (5)	−0.0013 (5)
C9	0.0299 (6)	0.0342 (7)	0.0432 (7)	0.0005 (5)	0.0003 (5)	−0.0024 (6)
C10	0.0320 (6)	0.0326 (6)	0.0362 (6)	0.0011 (5)	0.0026 (5)	−0.0045 (5)
C11	0.0309 (6)	0.0316 (6)	0.0347 (6)	0.0008 (5)	0.0007 (5)	−0.0020 (5)
C12	0.0308 (6)	0.0401 (7)	0.0387 (7)	−0.0002 (5)	−0.0007 (5)	−0.0031 (6)
C13	0.0365 (7)	0.0454 (8)	0.0388 (7)	0.0080 (6)	−0.0069 (6)	−0.0068 (6)
C14	0.0532 (9)	0.0428 (8)	0.0375 (7)	0.0086 (7)	−0.0030 (6)	0.0023 (6)
C15	0.0457 (8)	0.0391 (7)	0.0389 (7)	0.0001 (6)	0.0053 (6)	0.0023 (6)

### Geometric parameters (Å, º)

**Table:**

S1—C7	1.6587 (14)	C3—C4	1.355 (3)
F1—C3	1.361 (2)	C4—C5	1.379 (3)
O1—C9	1.2180 (17)	C5—C6	1.386 (2)
O2—N5	1.220 (2)	C8—C9	1.4974 (18)
O3—N5	1.222 (2)	C8—C11	1.4476 (18)
N1—C1	1.416 (2)	C10—C15	1.382 (2)
N1—C7	1.3411 (19)	C10—C11	1.4053 (18)
N2—N3	1.3434 (16)	C11—C12	1.3815 (18)
N2—C7	1.3823 (18)	C12—C13	1.385 (2)
N3—C8	1.2938 (17)	C13—C14	1.383 (2)
N4—C9	1.3679 (19)	C14—C15	1.385 (2)
N4—C10	1.3968 (17)	C2—H2	0.9300
N5—C13	1.466 (2)	C4—H4	0.9300
N1—H1	0.8600	C5—H5	0.9300
N2—H2A	0.8600	C6—H6	0.9300
N4—H4A	0.8600	C12—H12	0.9300
C1—C6	1.388 (2)	C14—H14	0.9300
C1—C2	1.384 (2)	C15—H15	0.9300
C2—C3	1.373 (3)		
			
C1—N1—C7	130.41 (13)	O1—C9—C8	126.90 (13)
N3—N2—C7	121.01 (11)	N4—C9—C8	105.98 (11)
N2—N3—C8	116.96 (11)	O1—C9—N4	127.11 (12)
C9—N4—C10	111.43 (11)	C11—C10—C15	121.88 (12)
O2—N5—O3	123.39 (16)	N4—C10—C11	109.13 (11)
O2—N5—C13	118.82 (14)	N4—C10—C15	128.99 (12)
O3—N5—C13	117.79 (14)	C8—C11—C12	132.17 (12)
C1—N1—H1	115.00	C8—C11—C10	107.09 (11)
C7—N1—H1	115.00	C10—C11—C12	120.74 (12)
N3—N2—H2A	119.00	C11—C12—C13	116.41 (12)
C7—N2—H2A	119.00	N5—C13—C12	117.56 (13)
C9—N4—H4A	124.00	N5—C13—C14	119.02 (14)
C10—N4—H4A	124.00	C12—C13—C14	123.42 (14)
N1—C1—C2	123.74 (14)	C13—C14—C15	120.15 (14)
N1—C1—C6	116.49 (14)	C10—C15—C14	117.40 (13)
C2—C1—C6	119.77 (14)	C1—C2—H2	121.00
C1—C2—C3	117.81 (16)	C3—C2—H2	121.00
F1—C3—C4	118.74 (17)	C3—C4—H4	121.00
C2—C3—C4	124.46 (18)	C5—C4—H4	121.00
F1—C3—C2	116.80 (17)	C4—C5—H5	119.00
C3—C4—C5	117.08 (18)	C6—C5—H5	119.00
C4—C5—C6	121.18 (17)	C1—C6—H6	120.00
C1—C6—C5	119.70 (16)	C5—C6—H6	120.00
S1—C7—N2	116.46 (11)	C11—C12—H12	122.00
S1—C7—N1	129.83 (11)	C13—C12—H12	122.00
N1—C7—N2	113.71 (12)	C13—C14—H14	120.00
C9—C8—C11	106.36 (11)	C15—C14—H14	120.00
N3—C8—C9	127.58 (12)	C10—C15—H15	121.00
N3—C8—C11	126.06 (12)	C14—C15—H15	121.00
			
C7—N1—C1—C2	0.8 (3)	C2—C3—C4—C5	−0.3 (3)
C7—N1—C1—C6	179.82 (15)	C3—C4—C5—C6	0.3 (3)
C1—N1—C7—S1	8.9 (2)	C4—C5—C6—C1	−0.3 (3)
C1—N1—C7—N2	−170.96 (14)	N3—C8—C9—O1	−0.1 (2)
C7—N2—N3—C8	−177.01 (12)	N3—C8—C9—N4	179.51 (13)
N3—N2—C7—S1	175.28 (10)	C11—C8—C9—O1	−179.74 (14)
N3—N2—C7—N1	−4.80 (18)	C11—C8—C9—N4	−0.17 (15)
N2—N3—C8—C9	−0.6 (2)	N3—C8—C11—C10	−179.13 (13)
N2—N3—C8—C11	179.00 (12)	N3—C8—C11—C12	0.3 (2)
C10—N4—C9—O1	179.28 (14)	C9—C8—C11—C10	0.57 (14)
C10—N4—C9—C8	−0.30 (15)	C9—C8—C11—C12	179.97 (16)
C9—N4—C10—C11	0.67 (16)	N4—C10—C11—C8	−0.76 (15)
C9—N4—C10—C15	−179.04 (14)	N4—C10—C11—C12	179.74 (12)
O2—N5—C13—C12	179.84 (15)	C15—C10—C11—C8	178.98 (13)
O2—N5—C13—C14	−0.1 (2)	C15—C10—C11—C12	−0.5 (2)
O3—N5—C13—C12	−0.5 (2)	N4—C10—C15—C14	179.89 (14)
O3—N5—C13—C14	179.53 (16)	C11—C10—C15—C14	0.2 (2)
N1—C1—C2—C3	178.66 (16)	C8—C11—C12—C13	−178.85 (14)
C6—C1—C2—C3	−0.3 (3)	C10—C11—C12—C13	0.5 (2)
N1—C1—C6—C5	−178.74 (15)	C11—C12—C13—N5	179.80 (13)
C2—C1—C6—C5	0.3 (2)	C11—C12—C13—C14	−0.2 (2)
C1—C2—C3—F1	−179.61 (16)	N5—C13—C14—C15	179.91 (14)
C1—C2—C3—C4	0.3 (3)	C12—C13—C14—C15	−0.1 (2)
F1—C3—C4—C5	179.64 (17)	C13—C14—C15—C10	0.1 (2)

### Hydrogen-bond geometry (Å, º)

**Table:**

*D*—H···*A*	*D*—H	H···*A*	*D*···*A*	*D*—H···*A*
N1—H1···N3	0.86	2.18	2.6166 (17)	112
N2—H2*A*···O1	0.86	2.06	2.7483 (16)	136
N4—H4*A*···O3^i^	0.86	2.26	3.0073 (17)	146
C2—H2···S1	0.93	2.49	3.1674 (17)	130
C4—H4···O1^ii^	0.93	2.39	3.281 (2)	161
C6—H6···O2^iii^	0.93	2.56	3.392 (2)	149
C12—H12···O2^iii^	0.93	2.59	3.470 (2)	158

Symmetry codes: (i) *x*−1/2, *y*, −*z*+1/2; (ii) *x*+1/2, −*y*+3/2, −*z*; (iii) −*x*+1, *y*+1/2, −*z*+1/2.
